# Early weight loss, diabetes remission and long-term trajectory after diagnosis of type 2 diabetes: a retrospective study

**DOI:** 10.1007/s00125-025-06402-w

**Published:** 2025-03-22

**Authors:** Mario Luca Morieri, Mauro Rigato, Vera Frison, Michele D’Ambrosio, Giovanni Sartore, Angelo Avogaro, Gian Paolo Fadini

**Affiliations:** 1 Department of Medicine, University of Padova, Padua, Italy; 2 Unit of Diabetology, Cittadella Hospital, ULSS6 Euganea, Cittadella, Italy; 3 Unit of Diabetology, Ospedali Riuniti Padova Sud, ULSS6 Euganea, Monselice, Italy; 4 Unit of Diabetology, Ospedale dei Colli, ULSS6 Euganea, Padua, Italy

**Keywords:** Complications, Glycaemic management, Remission, Type 2 diabetes, Weight management

## Abstract

**Aims/hypothesis:**

Weight loss can improve glycaemic management in individuals with type 2 diabetes, but its long-term effects on remission, cardiovascular risk factors and complications remain unclear. We investigated clinical outcomes following non-interventional ≥10% body weight loss in people with newly diagnosed type 2 diabetes in a routine care setting.

**Methods:**

We retrospectively analysed two cohorts of people with newly diagnosed type 2 diabetes. After exclusions, cohort 1 included 1934 individuals followed for up to 25 years; cohort 2 comprised 13,277 individuals followed for up to 10 years. Participants were categorised into two groups based on whether or not they lost at least 10% body weight. In a sensitivity analysis, a group of participants with intermediate weight loss (5% to <10%) was also considered. Outcomes included HbA_1c_, diabetes remission, cardiovascular parameters and chronic complications.

**Results:**

Participants (58% male) had a mean age of 62 years and a mean diabetes duration of <2 years at inclusion; mean baseline HbA_1c_ was 57–64 mmol/mol (7.4–8.0%) and mean BMI was ~30 kg/m^2^. Weight loss ≥10% was obtained in 15.9% (*n*=308) of participants in cohort 1 and in 8.8% (*n*=1167) in cohort 2. In cohort 1, weight loss ≥10% was associated with a sustained reduction in HbA_1c_ (mean difference 2.1 mmol/mol; 0.19%) and a higher remission rate than in the <10% weight loss group (20.2% vs 5.5%; HR 4.2). These findings were confirmed in cohort 2, with remission rates of 13.2% and 4.1% (HR 2.6) in the ≥10% and <10% weight loss groups, respectively. Weight loss ≥10% improved systolic BP and HDL-cholesterol and triglyceride levels. Participants with weight loss of 5% to <10% (28.2% in cohort 1 and 17.4% in cohort 2) had marginal improvements in HbA_1c_, lipids and remission rates compared with participants with weight loss <5%, and such results were inferior to those achieved with weight loss ≥10%. In cohort 1, compared with weight loss <5% (reference), the HR for remission was 5.2 with weight loss ≥10% vs 1.7 with weight loss 5% to <10%. Weight loss ≥10% was not associated with a reduced incidence of complications. On the other hand, remission was independently associated with a significantly lower rate of new-onset microangiopathy (adjusted HR 0.84; 95% CI 0.73, 0.97; *p*=0.019).

**Conclusions/interpretation:**

Early weight loss of ≥10% in type 2 diabetes was associated with sustained glycaemic improvements, increasing by three to four times the rates of diabetes remission. Remission, in turn, more than weight loss was associated with a reduced risk of complications.

**Graphical Abstract:**

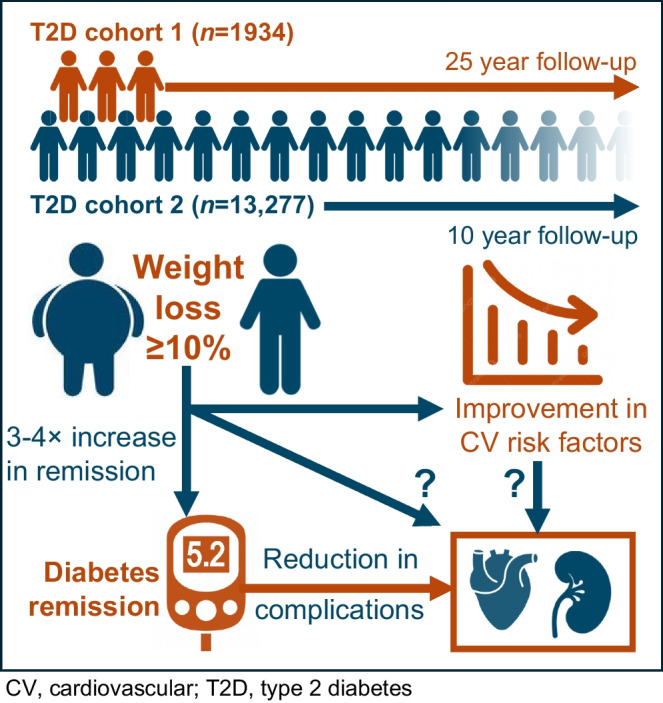

**Supplementary Information:**

The online version of this article (10.1007/s00125-025-06402-w) contains peer-reviewed but unedited supplementary material.


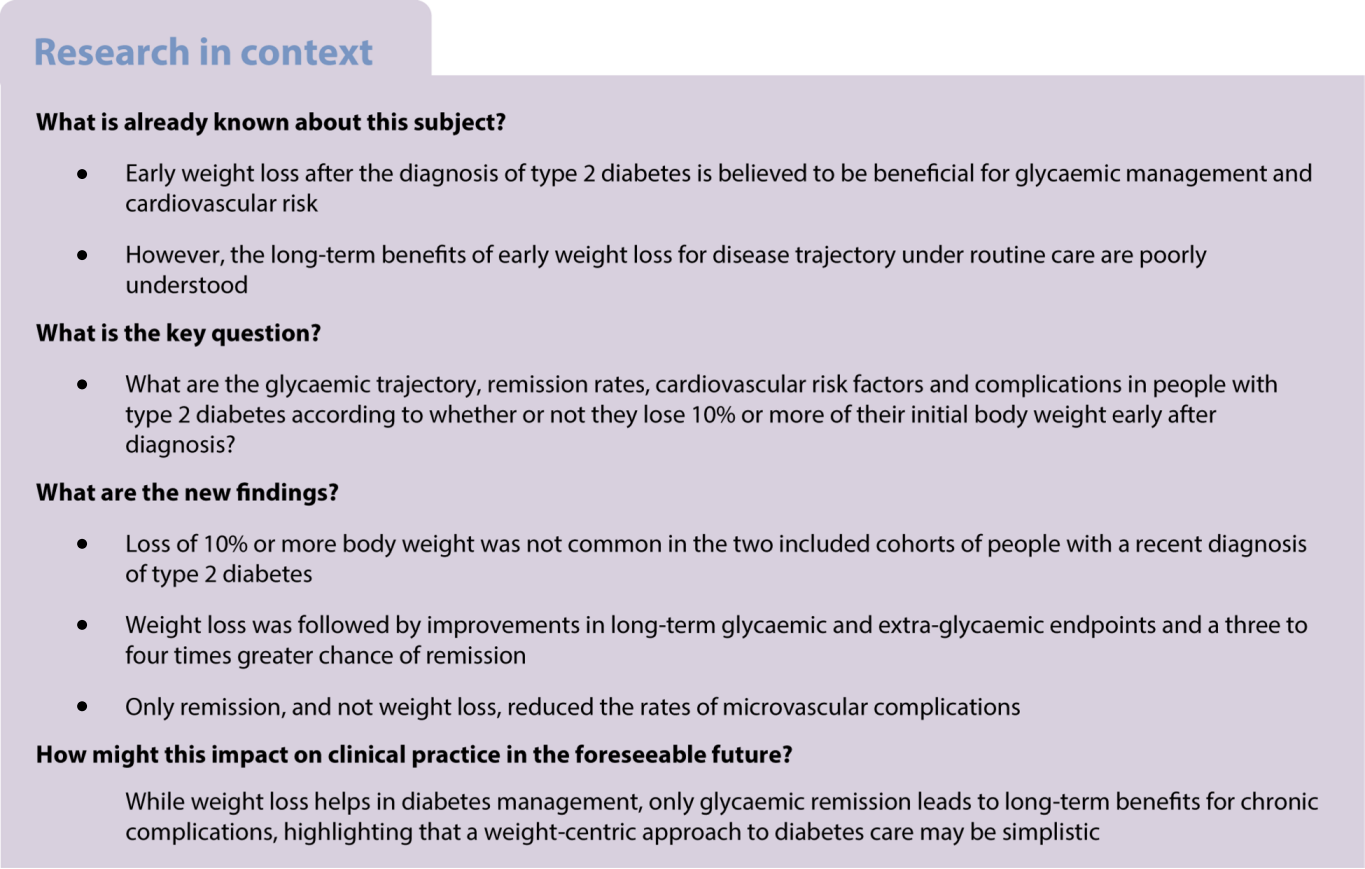


## Introduction

Type 2 diabetes features progressive hyperglycaemia and imposes a considerable burden due to chronicity and multi-organ complications. Early intervention through weight loss is crucial to improve blood glucose levels and cardiometabolic health and to mitigate complications [1]. Weight loss, especially early after diagnosis, may alter the natural course of type 2 diabetes by removing excess liver fat, improving insulin sensitivity and relieving beta cell stress, ultimately reducing the need for glucose-lowering medications (GLMs) [2].

Prominent evidence comes from the Look-AHEAD trial, investigating the effects of an intensive lifestyle intervention in type 2 diabetes. Participants in the intensive intervention group exhibited substantial glycaemic improvements, resulting in reduced reliance on GLMs and better cardiovascular health [3]. However, the benefit of the weight loss programme on blood glucose levels was much stronger in the short term, while resulting in a mean HbA_1c_ change of only −2.4 mmol/mol (−0.22%) over the 10 year observation period. The transient nature of the weight loss and cardiometabolic improvements highlights the challenges in maintaining long-term effects and explains the disappointing results for cardiovascular event rates. In an observational re-analysis of the trial, individuals who achieved ≥5% weight loss in the first year post diagnosis had a greater chance of achieving diabetes remission and exhibited lower glucose values over time [4].

A weight reduction ≥10% is often cited as the threshold for meaningful clinical improvements, possibly leading to diabetes remission [5]. Remission is currently defined as a value of HbA_1c_ <48 mmol/mol (<6.5%) at least 3 months after cessation of GLMs [6]. Given the short time frame of this definition, it is unclear whether remission leads to sustained improvements in metabolic health over extended periods or delays the progression of micro/macrovascular complications [7]. The 5 year follow-up of DiRECT found that a minority of participants with type 2 diabetes who achieved remission at 2 years after a weight management intervention persisted in the remission state [8]. The inconsistent long-term benefits seem to suggest that weight regain and the intrinsic progression of type 2 diabetes may offset the positive effects seen in the short term.

The analysis of large clinical databases may provide valuable information on this topic. However, there is a paucity of real-world studies reporting the long-term benefits of weight loss in people with type 2 diabetes under routine care and the associated outcomes. In this study, we analysed two large cohorts of newly diagnosed individuals with type 2 diabetes and identified those achieving ≥10% weight loss within 5 years after diagnosis. Our primary objective was to assess whether such weight loss was associated with long-term benefits for blood glucose levels, cardiometabolic parameters and disease burden.

## Methods

### Study design and objective

This was a retrospective multicentre study using routine clinical data and following the STROBE checklist. The diabetes outpatient clinic database in each centre was interrogated using MetaClinic (Me.Te.Da, Italy). Data were anonymised according to operative standards at the time of data extraction. The protocol was approved by the Ethical Committee of the University Hospital of Padua and informed consent was waived according to national regulations on retrospective studies using anonymised data. We aimed to evaluate whether early weight loss modified the glycaemic and metabolic trajectories of type 2 diabetes. To this end, we compared trends over time in glycaemic and cardiometabolic parameters of people with type 2 diabetes who lost ≥10% of their initial body weight within the first 5 years after the diagnosis of type 2 diabetes with those in people with type 2 diabetes who lost <10%.

### Cohorts and exposure

**Fig. 1:**
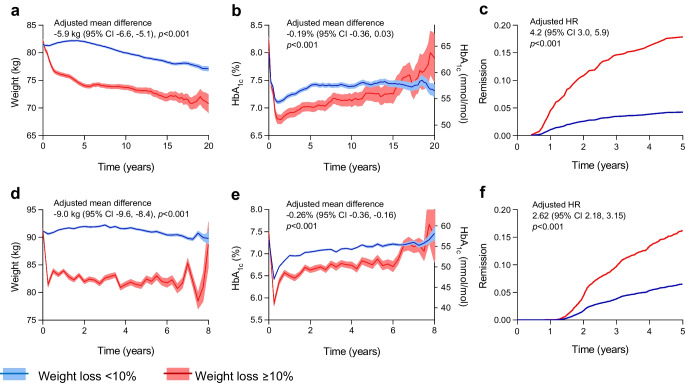
Main effects of weight loss. The figure compares selected endpoints between participants who lost ≥10% body weight and those who lost <10%. For cohort 1 (**a**–**c**) and cohort 2 (**d**–**f**), the panels show the change over time in body weight (**a**, **d**) and HbA_1c_ (**b**, **e**) analysed with the MMRM. (**c**) and (**f**) show the HRs of insulin initiation analysed with the Cox model. Adjusted effects are reported for each outcome along with the respective 95% CIs

The study was performed in two cohorts (electronic supplementary material [ESM] Fig. 1a). Cohort 1 included 11,254 individuals with type 2 diabetes, enrolled between 1992 and 2020 at the diabetes outpatient clinic of the University Hospital of Padua and observed for up to 25 years. Cohort 2 included 145,198 individuals with type 2 diabetes, enrolled between 2010 and 2018 at five diabetes outpatient clinics in the Veneto region (north-east Italy, population of ~2.3 million citizens) and observed for a maximum of 10 years. This population is representative of individuals with type 2 diabetes who attend diabetes clinics in Italy. In both populations, the date of type 2 diabetes diagnosis was collected as recorded in electronic records. We selected those who had a first evaluation within 2 years after diagnosis and had at least two evaluations of body weight within 5 years after diagnosis. Then, we retained only individuals who had at least one subsequent determination of HbA_1c_. Included participants were divided into two groups based on whether or not they lost ≥10% their initial body weight, which was the exposure. There was no constraint on the duration of weight loss. By design, all participants needed to be alive for at least 5 years after the diagnosis of type 2 diabetes in order to evaluate the exposure. The analysis was run in cohort 1 and then replicated in cohort 2. In a sensitivity analysis, we compared three groups of individuals with type 2 diabetes based on weight loss percentage: <5% (group 0, reference), 5% to <10% (group 1) and ≥10% (group 2).

### Variables

We collected the following variables: demographics (age, self-reported sex, date of diabetes diagnosis; race/ethnicity data were not available), anthropometrics (body weight, height, BMI), laboratory values (fasting glucose, HbA_1c_, total cholesterol and HDL-cholesterol, triglycerides, LDL-cholesterol [calculated with the Friedewald equation], serum creatinine for eGFR [using the Chronic Kidney Disease Epidemiology Collaboration (CKD-EPI) equation], urinary albumin/creatinine ratio [UACR]), comorbidities, diabetes complications and use of certain therapies. Data on complications were collected as recorded in electronic charts using ICD-9 codes (http://www.icd9data.com/2007/Volume1/default.htm) and standardised descriptions: retinopathy was based on digital fundus examination; chronic kidney disease (CKD) was based on eGFR and UACR; coronary artery disease was defined as a history of acute coronary syndrome or coronary revascularisation; cerebrovascular disease was defined as stroke or transient ischaemic attack or cerebral revascularisation; CVD was defined as a history of coronary or cerebral events or any-site revascularisation; macrovascular disease included asymptomatic atherosclerosis; and microvascular disease included any of retinopathy, CKD or neuropathy. Comorbidities included cancer, history of inflammatory disease and a comorbidity index (Rx-Risk). Among therapies, we considered GLM classes (metformin, sulfonylureas, dipeptidyl peptidase 4 [DPP-4] inhibitors, glucagon-like peptide-1 receptor agonists [GLP-1RAs], sodium−glucose cotransporter 2 [SGLT2] inhibitors, acarbose, thiazolidinediones and insulin) and therapies for the management of cardiovascular risk factors.

### Outcomes

The primary outcome was the change in HbA_1c_. Secondary outcomes were computed for participants with available data and included remission of diabetes (defined as two consecutive HbA_1c_ values <48 mmol/mol [<6.5%] at least 90 days apart off therapy) within 5 years after diagnosis; change in other continuous variables (BP, lipids, eGFR, UACR); number of GLMs; and probability of initiating insulin. New microangiopathy (retinopathy, neuropathy, CKD or UACR >3.4 mg/mmol [30 mg/g]) and new macroangiopathy (myocardial infarction, stroke, or coronary, cerebral or peripheral arterial revascularisation) were exploratory outcomes. Complete data on mortality rate were not available and participants were included in the analysis for as long as they were observed in the diabetes specialist database, with no possibility of distinguishing between loss to follow-up, referral to a family doctor or death.

### Statistical analysis

Continuous variables are reported as mean (SD), whereas categorical variables are reported as percentages. The difference between two groups was examined with Student’s *t* test or χ^2^ test for continuous or categorical variables, respectively. Non-normal variables were log-transformed before analysis with parametric tests. Variables independently associated with ≥10% weight loss or remission were identified by a logistic multiple regression model. The change over time in continuous variables was examined using the mixed model for repeated measures (MMRM): dependent variables were the outcome variables described above; fixed factors were time, group, time-by-group interaction and the baseline values of the dependent variables; covariates were those that differed significantly between groups on univariate analysis. To detect short- and long-term effects, the analysis of cohort 1 was run for the entire observation period (20 years) and for the first 5 years. The occurrence of events during follow-up was compared using the Cox proportional hazards model. Statistical significance was set at *p*<0.05 without adjusting for the multiplicity of testing. The statistical packages SPSS version 23 (IBM, Armonk, NY, USA) and SAS version 9.4 (SAS Institute, Cary, NC, USA) were used.

## Results

### Characteristics of cohort 1

Of the 11,254 individuals in cohort 1 (ESM Fig. 1b), 3970 had a first evaluation within 2 years after diagnosis, and 3902 had available data to determine exposure (at least two body weight measurements within 5 years after diagnosis). We retained 1934 participants with available data for the primary outcome (change in HbA_1c_), who were divided into those who lost ≥10% of their initial body weight (*n*=308; 15.9%) and those who did not (*n*=1626). Mean age was 62.7 years and 42.7% of participants were female, mean baseline BMI was 29.5 kg/m^2^ and mean HbA_1c_ was 64 mmol/mol (8.0%). The mean known diabetes duration was 0.8 years. No participants underwent bariatric surgery. The two groups significantly differed for sex, baseline BMI, HbA_1c_, degree of comorbidities and use of metformin, sulfonylurea and insulin (Table 1). The median (IQR) duration of observation was 10.8 (7.3–15) years, with a maximum of 24.8 years. The main characteristics of individuals who were excluded because of missing data are shown in ESM Table 1.

**Table 1:** Baseline characteristics of the study cohorts

Characteristic	Cohort 1				Cohort 2			
	All	Weight loss ≥10%	Weight loss <10%	*p* value	All	Weight loss ≥10%	Weight loss <10%	*p* value
Number	1934	308	1626		13,277	1167	12,110	
Male	57.3	49.7	58.7	0.003	58.9	49.3	59.8	<0.001
Age, years	62.7 (11.7)	62.0 (12.5)	62.9 (11.5)	0.218	61.6 (12.7)	61.4 (13.2)	61.6 (12.6)	0.551
Diabetes duration, years	0.8 (1.8)	0.8 (1.8)	0.8 (1.8)	0.803	1.0 (1.3)	0.5 (1.0)	1.0 (1.4)	<0.001
BMI, kg/m^2^	29.5 (5.2)	31.1 (5.8)	29.2 (5.0)	<0.001	30.1 (5.7)	32.0 (6.4)	29.9 (5.6)	<0.001
Laboratory and risk factors								
HbA_1c_				0.050				<0.001
mmol/mol	64 (17)	66 (17)	64 (17)		57 (13)	59 (14)	56 (13)	
%	8.0 (2.1)	8.2 (2.1)	8.0 (2.1)		7.4 (1.7)	7.6 (1.8)	7.3 (1.7)	
Systolic BP, mmHg	140.5 (20.3)	141.2 (21.7)	140.4 (20.0)	0.542	137.8 (19.4)	139.2 (20.5)	137.7 (19.3)	0.017
Diastolic BP, mmHg	83.1 (10.7)	83.9 (11.8)	83.0 (10.5)	0.173	80.7 (10.6)	81.3 (11.0)	80.7 (10.6)	0.095
Total cholesterol				0.507				<0.001
mmol/l	5.4 (1.3)	5.5 (1.4)	5.4 (1.3)		5.0 (1.1)	5.1 (1.2)	5.0 (1.1)	
mg/dl	206.8 (48.3)	208.8 (51.5)	206.5 (47.7)		189.1 (43.3)	193.6 (44.0)	188.7 (43.2)	
HDL-cholesterol				0.586				0.113
mmol/l	1.3 (0.4)	1.3 (0.4)	1.3 (0.4)		1.3 (0.4)	1.3 (0.4)	1.3 (0.4)	
mg/dl	49.8 (14.9)	49.3 (14.2)	49.9 (15.0)		49.0 (13.7)	48.3 (14.2)	49.1 (13.7)	
Triglycerides				0.104				0.002
mmol/l	1.7 (0.9)	1.8 (1.0)	1.7 (0.8)		1.7 (1.3)	1.8 (1.4)	1.7 (1.2)	
mg/dl	150.0 (77.0)	158.6 (91.1)	148.4 (74.0)		148.0 (112.2)	158.7 (120.7)	147.0 (111.3)	
LDL-cholesterol				0.734				0.007
mmol/l	3.3 (1.1)	3.3 (1.1)	3.3 (1.1)		2.9 (1.0)	3.0 (1.0)	2.9 (1.0)	
mg/dl	124.5 (39.6)	123.6 (41.3)	124.7 (39.3)		110.7 (36.9)	113.8 (37.0)	110.4 (36.8)	
eGFR, ml/min per 1.73 m^2^	81.0 (19.1)	82.3 (21.3)	80.8 (18.7)	0.308	83.7 (22.0)	83.2 (21.6)	83.7 (22.0)	0.440
UACR				0.219				0.265
mg/g	4.9 (15.5)	2.9 (6.3)	5.3 (16.7)		5.8 (25.3)	7.4 (29.7)	5.7 (24.9)	
mg/mmol	43.0 (137.2)	25.6 (56.1)	46.6 (148.2)		51.6 (223.9)	65.3 (263.0)	50.4 (219.9)	
Complications								
eGFR <60 ml/min per 1.73 m^2^	14.1	14.4	14.0	0.865	10.3	11.0	10.2	0.398
UACR >3.4 mg/mmol (30 mg/g)	20.0	16.7	20.9	0.398	5.3	6.3	5.3	0.149
Retinopathy	18.7	13.8	19.4	0.473	1.3	0.9	1.4	0.148
CVD	2.4	2.9	2.3	0.495	2.6	2.7	2.6	0.826
Microvascular disease	37.9	35.9	38.2	0.622	16.4	17.7	16.3	0.240
Macrovascular disease	4.2	4.2	4.2	0.975	7.4	5.1	7.6	0.001
Comorbidities								
History of cancer	5.7	8.1	5.2	0.045	N/A	N/A	N/A	N/A
Inflammatory disease	12.2	17.2	11.2	0.003	N/A	N/A	N/A	N/A
Rx-Risk index	4.7 (5.1)	5.8 (6.5)	4.5 (4.8)	<0.001	4.0 (5.4)	4.5 (6.0)	3.9 (5.3)	<0.001
Diabetes therapy								
Metformin	43.6	50.3	42.4	0.010	50.5	50.4	50.5	0.930
Sulfonylurea	24.3	16.2	25.8	<0.001	6.7	5.7	6.8	0.142
DPP-4 inhibitors	1.8	2.9	1.5	0.090	3.5	2.2	3.6	0.013
GLP-1RAs	0.7	0.6	0.7	0.866	0.7	0.9	0.7	0.354
SGLT2 inhibitors	0.3	0.6	0.2	0.141	0.6	0.5	0.6	0.707
Pioglitazone	0.3	0.0	0.4	0.286	0.8	0.7	0.8	0.757
Bolus insulin	11.1	7.1	11.9	0.016	4.6	5.0	4.5	0.504
Basal insulin	6.7	6.8	6.7	0.941	4.9	3.8	5.0	0.055
Other therapies								
Statins	25.6	26.9	25.3	0.553	37.8	33.9	38.2	0.004
Anti-platelet agents	51.8	54.2	51.3	0.346	25.8	22.3	26.1	0.004
RAS blockers	41.0	43.2	40.6	0.397	45.0	43.7	45.2	0.333
β-blockers	16.6	17.9	16.4	0.517	20.3	21.2	20.3	0.465
Calcium channel blockers	16.6	16.9	16.6	0.904	15.0	14.1	15.1	0.360
Diuretics	28.1	32.1	27.3	0.083	29.2	29.1	29.2	0.958

Data are presented as mean (SD) or percentage

N/A, not applicable; RAS, renin–angiotensin system

### Weight and glycaemic trajectory

After adjusting for baseline weight in the model, the weight curves diverged early by design and remained separated for up to 20 years (Fig. 1a). The difference was greater at 5 years (~8 kg) and the total adjusted mean difference was 5.9 kg (95% CI 5.1, 6.6). In logistic regression analysis, ≥10% weight loss was associated with female sex, higher BMI, not being on sulfonylurea or insulin, and comorbidity status (ESM Table 2a).

From initial values of 64–66 mmol/mol (8.0–8.2%), HbA_1c_ declined rapidly in the first year, reaching 51 mmol/mol (6.8%) and 55 mmol/mol (7.2%) in the ≥10% and <10% weight loss groups, respectively (Fig. 1b). The two curves remained separated for at least 10 years and the adjusted mean difference was 2.1 mmol/mol (95% CI 0.3, 3.9 [0.19%; 95% CI 0.03%, 0.36%]). The difference was greater during the first 5 years (3.4 mmol/mol [0.31%]; ESM Table 3).

The burden of therapy (number of GLM classes) increased steadily in the two groups (ESM Fig. 2a), but the ≥10% weight loss group had a marginally lower burden of therapy by 0.2 drug classes (*p*=0.002). Among participants who were insulin-free at baseline, 21.4% initiated insulin and there was no significant difference in the adjusted rate of insulin initiation between groups (HR 1.22; 95% CI 0.92, 1.61; *p*=0.165; ESM Fig. 2c).

Remission was achieved by 145 individuals, 20.2% in the group with ≥10% weight loss and 5.5% in the group with <10% weight loss (adjusted HR 4.2; 95% CI 3.0, 5.9; Fig. 1c). The median (IQR) duration of remission was 1.8 (0.9–3.4) years and was similar in the two groups.

### Cardiometabolic parameters and complications

Systolic BP declined early and remained significantly lower for up to 20 years in the ≥10% weight loss group, with a difference that was more evident during the first 5 years (ESM Fig. 3a). Triglyceride levels declined early in the ≥10% weight loss group and the mean adjusted difference vs the group with <10% weight loss in the first 5 years was −0.13 mmol/l (−11.2 mg/dl; *p*<0.001; ESM Fig. 3c), but the difference was lost on longer observation. HDL-cholesterol remained significantly higher by 0.04 mmol/l (1.4 mg/dl) in the ≥10% weight loss group for up to 20 years (ESM Fig. 3d). LDL-cholesterol declined in both groups, but was higher (by 0.18 mmol/l; 6.8 mg/dl) in the ≥10% weight loss group, as was total cholesterol (ESM Table 3).

eGFR (median of 11 values per participant) increased early in the ≥10% weight loss group and remained higher by 2.4 ml/min per 1.73 m^2^ (ESM Fig. 4a). The change in UACR was highly variable and did not differ between groups (ESM Fig. 4c), but only a median of four values per participant was available. The rate of new-onset CKD, examined in those who were free from CKD at baseline, did not differ between groups (adjusted HR 0.93; 95% CI 0.79, 1.24; *p*=0.928).

The incidence of new micro- or macroangiopathy was evaluated in participants who were free from these complications at baseline (*n*=1561 and *n*=1810, respectively). During observation, 55.1% and 55.5% developed new micro- or microangiopathy, respectively. There was no difference between groups in the rates of incident micro- or macroangiopathy (adjusted HR 1.05 and 1.01, respectively).

### Replication in cohort 2

Of 145,198 individuals with type 2 diabetes in cohort 2, 50,080 had available data within 2 years after diagnosis of type 2 diabetes, 29,536 had body weight data to assess exposure and 13,277 had outcome data (ESM Fig. 1b). Participants had a mean age of 61.6 years and 58.9% were male; mean baseline BMI was 30.1 kg/m^2^ and mean HbA_1c_ was 57 mmol/mol (7.4%). The median (IQR) duration of diabetes at the beginning of observation was 0 (0–2) years. Weight loss of ≥10% was achieved by 1167 participants (8.8%). The two groups differed significantly for sex, diabetes duration, baseline BMI, HbA_1c_, lipids, prevalence of macroangiopathy, comorbidity index and therapy with DPP-4 inhibitors, statins and anti-platelet agents (Table 1). The median (IQR) observation period was 4.0 (2.0–6.3) years.

The mean adjusted difference in body weight between the groups was 9.0 kg (95% CI 8.4, 9.6; Fig. 1d). Variables significantly associated with weight loss were female sex, shorter diabetes duration, higher BMI, HbA_1c_ and comorbidity index (ESM Table 2b).

HbA_1c_ declined more in the ≥10% weight loss group and remained lower by 2.8 mmol/mol (0.26%) than in the <10% weight loss group, but the curves converged after 6 years (Fig. 1e). The rates of remission were 13.2% in the group with weight loss ≥10% and 4.1% in the group with <10% weight loss (adjusted HR 2.62; 95% CI 2.18, 3.15; Fig. 1f). The median (IQR) duration of remission was 1.3 (0.6–2.1) years and was similar in the two groups.

The number of GLM classes used by the participants increased similarly in the two groups (ESM Fig. 2b) and 4.5% in the two groups combined initiated insulin. The ≥10% weight loss group had a significantly greater risk of initiating insulin (HR 1.76; 95% CI 1.39, 2.25; *p*<0.001; ESM Fig. 2d).

In the ≥10% weight loss group, there were significantly greater improvements in systolic BP (ESM Fig. 3b), serum triglycerides (ESM Fig. 3d) and HDL-cholesterol (ESM Fig. 3f), while LDL-cholesterol declined similarly (ESM Table 3).

No significant differences were observed in the trends over time in eGFR and UACR (ESM Fig. 4b, d).

During observation, 33.0% developed a new microangiopathy (yearly incidence 8.2%) and 23.9% developed a new macroangiopathy (yearly incidence 6.0%) among participants who were free from these complications at baseline. The incidence rates of new micro- and macroangiopathy were similar between the two groups (Fig. 3).

### Features and consequences of remission

**Fig. 2:**
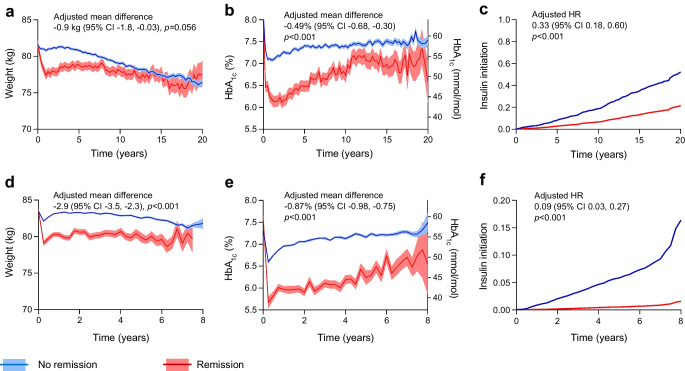
Main effects of diabetes remission. The figure compares selected endpoints between participants with diabetes remission and those without. For cohort 1 (**a**–**c**) and cohort 2 (**d**–**f**), the panels show the change over time in body weight (**a**, **d**) and HbA_1c_ (**b**, **e**) analysed with the MMRM. (**c**) and (**f**) show the HRs of insulin initiation analysed with the Cox model. Adjusted effects are reported for each outcome along with the respective 95% CIs

In cohort 1, remission was associated with shorter diabetes duration, lower prevalence of macrovascular disease and lower use of metformin and sulfonylurea. In cohort 2, remission was associated with shorter disease duration, lower BMI, higher BP and HbA_1c_, lower rates of macroangiopathy and lower use of metformin, sulfonylureas, DPP-4 inhibitors, insulin and statins (ESM Table 4). HbA_1c_ and body weight trends in those with and without remission are shown in Fig. 2a, b (cohort 1) and Fig. 2d, e (cohort 2).

In both cohorts, remission led to a marked reduction in insulin initiation (cohort 1: adjusted HR 0.33; 95% CI 0.18, 0.60; Fig. 2c; cohort 2: adjusted HR 0.09; 95% CI 0.03, 0.27; Fig. 2f).

**Fig. 3:**
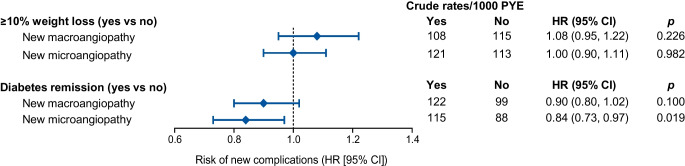
Rates of complications. The forest plot reports crude rates of new microangiopathy and new macroangiopathy in the groups indicated. HRs with 95% CIs and *p* values are also shown. PYE, patient-years of exposure

We evaluated the association between remission and occurrence of new complications in cohort 2 (Fig. 3). In the unadjusted analysis, individuals who achieved remission had significantly lower rates of microangiopathy (HR 0.77; 95% CI 0.65, 0.90; *p*=0.001) and macroangiopathy (HR 0.84; 95% CI 0.73, 0.96; *p*=0.011). When adjusted for clinical variables significantly associated with remission, the protection from new microangiopathy was still evident (HR 0.84; 95% CI 0.73, 0.97; *p*=0.019), whereas significance was lost for macroangiopathy (HR 0.90; 95% CI 0.80, 1.02; *p*=0.100).

### Sensitivity analyses

We evaluated mean weight loss by remission status in the two groups. ESM Fig. 5 shows that diabetes remission in the group with <10% weight loss was associated with small weight differences. Within the ≥10% weight loss group, remission was associated with a faster weight loss in the first 2 years, but the difference was lost in the long run.

Finally, for both cohorts, we performed a three-group comparison based on weight loss: <5% (group 0); 5% to <10% (group 1); and ≥10% (group 2; ESM Tables 5, 6). Compared with group 0, group 1 (28.2% in cohort 1 and 17.4% in cohort 2) demonstrated a significant but marginal improvement in HbA_1c_ (mean adjusted difference −1.3 mmol/mol [−0.12%] in cohort 1 and −1.4 mmol/mol [−0.13%] in cohort 2; ESM Table 7), which was inferior to that observed in group 2 and which was no longer present after 8–10 years (ESM Fig. 6b, e). Remission was strikingly more frequent in group 2 than in group 1, especially in cohort 1 (HR vs group 0: 5.2 and 1.7, respectively; ESM Fig. 6c). BP and lipids improved marginally in group 1 and significantly more in group 2 than in group 1 (ESM Table 7).

## Discussion

Our findings from two independent cohorts of people with newly diagnosed type 2 diabetes underscore the beneficial impact of ≥10% early weight loss on several clinical outcomes, including HbA_1c_, cardiovascular risk factors and diabetes remission.

Individuals in the ≥10% weight loss group exhibited a more rapid decline in HbA_1c_ in the first year, reaching significantly lower levels than individuals in the <10% weight loss group. The early divergence of HbA_1c_ curves between the groups suggests that substantial weight loss can exert an immediate impact on glucose metabolism, possibly due to restored beta cell function, enhanced insulin sensitivity and reduced hepatic glucose output [9].

Cardiovascular risk factors (systolic BP, HDL-cholesterol and triglycerides) were significantly improved in the group with ≥10% weight loss, particularly in the first 5 years of follow-up. This is consistent with available literature demonstrating the cardiovascular benefits of weight loss in type 2 diabetes [3, 4, 10, 11].

One of the most important findings of this study was the association between weight loss and diabetes remission. In cohort 1, individuals in the ≥10% weight loss group were four times more likely to achieve remission than those in the <10% weight loss group (20.2% vs 5.5%). These findings were replicated in cohort 2, with remission rates of 13.2% vs 4.1%, respectively. Participants who achieved remission had a markedly lower hazard of initiating insulin, emphasising the clinical importance of weight loss in reducing treatment burden and potentially modifying the natural history of diabetes. Despite weight loss being associated with a three- to fourfold increase in remission, remission rates were relatively low compared with those observed after metabolic/bariatric surgery [12]. In the Look-AHEAD trial, with a mean 8% lower body weight in the intensive lifestyle intervention group, the rate of remission was 11.5% [4]. In the real-world implementation of a weight management programme by the English National Health Service (NHS), which found a mean weight loss of 8.3% (9.4 kg), remission was achieved in 27% of participants with type 2 diabetes diagnosed within 6 years [13]. An observational study performed in Hong Kong recently highlighted that the incidence of diabetes remission (6% within 8 years after diagnosis) and its persistence were both low with conventional real-world management [14]. Therefore, remission rates in our study are in line with the literature. Duration of remission in cohort 1 was similar to that observed in DiRECT [8], with only ~10% of initial participants persisting in remission after ~5 years.

The analysis of three groups based on weight loss provides further validation of the 10% cut-off for greater glycaemic improvements, likelihood of remission and extra-glycaemic benefits, supporting international recommendations on weight management in type 2 diabetes [15].

Although weight loss was sustained over time, with favourable effects on blood glucose levels and cardiometabolic parameters, there were no significant differences in the incidence of new micro- or macroangiopathy. This may be partly explained by the relatively high rates of these complications in both groups, reflecting the progressive nature of type 2 diabetes. While weight loss may improve intermediate markers of cardiovascular health, the long-term incidence of diabetes-related complications could be influenced by other factors. For microangiopathy, the entity of HbA_1c_ reduction may have been too small and transient to reduce hyperglycaemic damage. For macroangiopathy, the higher levels of LDL-cholesterol in the weight loss group may have offset the improvements of other cardiometabolic markers. This finding parallels the primary results of the Look-AHEAD trial, where there was no between-group difference in the occurrence of the primary cardiovascular endpoint [3].

On the other hand, we found that participants who achieved diabetes remission, even if transient, had lower unadjusted rates of micro-/macroangiopathy, which remained significant for microangiopathy after adjusting for confounders. Protection against microvascular complications likely relies on a reduced cumulative exposure to hyperglycaemia. Similar results were achieved for remission after bariatric surgery in type 2 diabetes [16, 17] and are in line with secondary results of the Look-AHEAD study, reporting that participants with diabetes remission had a substantially lower incidence of CKD and CVD [18]. The loss of significance for macroangiopathy in our adjusted model indicates that drivers of remission partially explain the subsequent protection against cardiovascular complications.

A major strength of this study is the use of two large, well-characterised cohorts with long-term follow-up and extensive data granularity. The consistency of findings across cohorts enhances the external validity of our results, particularly given the real-world nature of the data.

However, some limitations should be noted. First, the observational design precludes causal inferences, and confounding factors may influence the associations between weight loss and outcomes. In non-interventional real-world studies, it is hard to recall the cause of weight loss. While the use of GLP-1RAs and SGLT2 inhibitors was minimal and no participants underwent bariatric surgery in cohort 1, it remains unknown whether weight loss was voluntary or associated with intercurrent diseases. This leaves open the possibility of reverse causality confounding the interpretation of outcome results [19], as acute conditions or cancer can eventually worsen long-term prognosis. Participants needed to be alive for 5 years after diagnosis, thereby reasonably ruling out weight loss due to advanced cancer, but other confounding events could not be controlled for. We also acknowledge that we had no data on dietary habits or physical exercise.

Second, the study relied on routinely collected clinical data, which may be subject to measurement errors or incomplete follow-up. Also, the long-term changes in clinical parameters in a real-world setting cannot be entirely attributed to initial weight loss, as changes in medications and the onset of new medical conditions can exert a major role. A striking example is the reduction in LDL-cholesterol over time, reflecting the implementation of LDL-cholesterol target recommendations [20].

Third, the relatively small proportion of participants achieving substantial weight loss, particularly in cohort 2, limits the power to detect differences in some outcomes. As a note of caution, we underline the lack of a link to administrative data on chronic complications, which are therefore subject to reporting bias and were considered exploratory endpoints, including because competing death could not be analysed.

Finally, the generalisability of the findings warrants scrutiny, as the data were collected under diabetes specialist care settings and may not apply elsewhere. Many individuals were excluded due to missing data and, without assuming missing-at-random, it is impossible to determine whether included and excluded individuals truly differed in key features that could influence the outcome.

Despite these limitations, our study conveys clinically relevant messages. Considering that remission could occur even with minimal weight change, it is noteworthy that remission, more than weight loss, was necessary for long-term benefits for complications. We observed that body weight and HbA_1c_ are interconnected but followed different long-term trajectories. This highlights that weight loss can influence HbA_1c_ but may not be sufficient to sustain long-term remission, as the progressive nature of type 2 diabetes likely erodes early benefits unless additional strategies are implemented to mitigate beta cell dysfunction. In addition to increasing the likelihood of remission, the effects on BP and lipids further support the integration of weight loss strategies into routine diabetes care, but only remission was able to modify disease trajectory, therapeutic burden and future complications. In the SURMOUNT-1 trial, a 19% weight loss obtained with tirzepatide almost nullified the progression from prediabetes to type 2 diabetes in people with obesity. However, after drug discontinuation, there was a striking rebound in the rates of progression to type 2 diabetes, despite weight maintenance for 3 years [21]. Thus, beta cell failure and the intrinsic tendency to progression appear to take over even in the presence of marked weight efficacy. Future research should address the mechanisms underlying the attenuation of glycaemic benefits in the long run after weight loss and strategies to sustain remission.

In conclusion, our findings suggest that significant weight loss in the early stages of type 2 diabetes can rapidly improve glucose management and cardiometabolic parameters, strongly increasing the rates of remission. However, remission, but not weight loss per se, impacted on disease trajectory and long-term diabetes complications, underscoring the need for comprehensive diabetes care beyond weight management.

## Supplementary Information

Below is the link to the electronic supplementary material.

ESM (PDF 1034 KB)

## Data Availability

Restrictions apply to the sharing of rough data used for this study. Aggregate data are available on reasonable request to the corresponding author.

## Abbreviations

CKD: Chronic kidney disease
DPP-4: Dipeptidyl peptidase 4
GLM: Glucose-lowering medication
GLP-1RA: Glucagon-like peptide-1 receptor agonist
MMRM: Mixed model for repeated measures
SGLT2: Sodium−glucose cotransporter 2
UACR: Urinary albumin/creatinine ratio
