# Supplementary material for: Early weight loss, diabetes remission and long-term trajectory after diagnosis of type 2 diabetes: a retrospective study
Source: Diabetologia. 2025 Mar 22;68(6):1115–25. doi: 10.1007/s00125-025-06402-w (PMC12069414; doi:10.1007/s00125-025-06402-w)

Electronic Supplemental Material

ESM Tab. 1. Main characteristics of included versus excluded individuals in both cohorts. As participants were excluded form the analysis because of missing data, a formal comparison was not possible and only key characteristics are shown for a qualitative evaluation of generalizability.

	**Cohort 1**		**Cohort 2**	
	**Included**	**Excluded**	**Included**	**Excluded**
**Number**	1934	2036	13,277	36,803
**Age, years**	62.7 (11.7)	62.8 (12.8)	61.6 (12.7)	65.7 (13.3)
**Male sex, %**	57.3	58.9	58.9	58.5
**Diabetes duration, years**	0.8 (1.8)	0.3 (1.6)	1.0 (1.3)	1.3 (1.4)
**Body mass index, kg/m^2**	29.5 (5.2)	29.2 (5.3)	30.1 (5.7)	30.0 (5.8)
**HbA_{1c}, mmol/mol**<br>**%**	64 (17)<br>8.0 (2.1)	69 (19)<br>8.5 (2.3)	57 (13)<br>7.4 (1.7)	54 (11)<br>7.1 (1.5)
**Total cholesterol, mg/dl**<br>**mmol/l**	206.8 (48.3)<br>5.3 (1.7)	209.3 (54.6)<br>5.4 (1.9)	189.1 (43.3)<br>4.8 (1.5)	182.2 (44.9)<br>4.7 (1.5)
**Triglycerides, mg/dl**<br>**mmol/l**	150.0 (77.0)<br>1.7 (0.9)	156.6 (84.4)<br>1.8 (0.9)	148.0 (112.2)<br>1.7 (1.3)	147.9 (105.7)<br>1.7 (1.2)
**eGFR, ml/min/1.73 m^2**	81.0 (19.1)	81.9 (21.8)	83.7 (22.0)	81.0 (23.7)
**Complications**				
**Microvascular disease, %**	37.9	50.5	16.4	21.0
**Macrovascular disease, %**	4.2	4.4	7.4	36.0
**Diabetes therapy**				
**Metformin, %**	43.6	42.0	50.5	28.1
**Sulphonylurea, %**	24.3	24.2	6.7	3.4
**Bolus insulin, %**	11.1	17.3	4.6	3.0
**Basal insulin, %**	6.7	13.2	4.9	3.9

ESM Tab. 2. Variables independently associated with weight loss ≥10%. The table shows results or a logistic regression analysis, where the dependent variable was the weight loss ≥10% and independent variables were those differing upon univariate analysis.

a) Cohort 1

Variable	OR (95% C.I.)	p
**Female sex**	1.34 (1.04; 1.72)	0.024
**BMI**	1.06 (1.03; 1.08)	<0.001
**HbA_{1c}**	1.11 (1.04; 1.18)	<0.001
**Use of sulphonylurea**	0.52 (0.37; 0.73)	<0.001
**Use of metformin**	1.12 (0.86; 1.47)	0.400
**Use of bolus insulin**	0.39 (0.23; 0.65)	<0.001
**History of cancer**	1.78 (1.09; 2.87)	0.021
**Inflammatory disease**	1.62 (1.14; 2.31)	0.007
**RxRisk comorbidity index**	1.04 (1.02; 1.07)	<0.001

b) Cohort 2

Variable	OR (95% C.I.)	p
**Female sex**	1.42 (1.26; 1.61)	<0.001
**Diabetes duration**	0.69 (0.65; 0.73)	<0.001
**BMI**	1.04 (1.03; 1.05)	<0.001
**HbA_{1c}**	1.05 (1.02; 1.09)	0.002
**Total cholesterol**	1.00 (0.99;1.00)	0.581
**Macroangiopathy**	1.16 (0.87; 1.55)	0.318
**RxRisk index**	1.02 (1.01; 1.03)	0.001
**DPP-4 inhbiitors**	0.88 (0.59; 1.33)	0.546
**Statins**	0.97 (0.84; 1.12)	0.655
**Anti-platelet agents**	0.88 (0.74; 1.04)	0.131

ESM Tab. 3. Outcomes. Differences between groups in the change over time of continuous outcome variables. Results are from adjusted MMRM. Negative values indicate a reduction in the weight loss versus the control group.

	Cohort 1 (5 years)		Cohort 1 (20 years)		Cohort 2	
	**Difference (95% CI)**	**p-value**	**Difference (95% CI)**	**p-value**	**Difference (95% CI)**	**p-value**
Primary outcome						
HbA1c, mmol/mol<br>%	-3.4 (-4.5, 2.4)<br>-0.31 (-0.41, -0.22)	<0.001	-2.1 (-3.9, -0.3)<br>-0.19 (-0.36, 0.03)	<0.001	-2.8 (-3.9, -1.7)<br>-0.26 (-0.36, -0.16)	<0.001
Secondary outcomes						
No. GLM classes	0.03 (-0.05, 0.11)	0.470	-0.2 (-0.36, -0.07)	0.004	-0.05 (-1.0, 0.004)	0.072
Systolic blood pressure, mm Hg	-2.7 (-3.7, -1.7)	<0.001	-1.9 (-3.8, -0.03)	0.047	-4.4 (-6.2, -2.6)	<0.001
HDL cholesterol, mg/dl<br>mmol/l	1.4 (0.4, 2.3)<br>0.04 (0.01, 0.06)	0.005	1.4 (-0.1, 2.9)<br>0.04 (-0.00, 0.07)	0.071	2.1 (1.3, 2.9)<br>0.05 (0.03, 0.07)	<0.001
Triglycerides, mg/dl<br>mmol/l	-11.2 (-17.2, -5.3)<br>-0.1 (-0.2, -0.1)	<0.001	-0.9 (-9.6, 7.7)<br>0.0 (-0.1, 0.1)	0.833	-16.7 (-23.4, -10.1)<br>-0.2 (-0.3, -0.1)	<0.001
LDL cholesterol, mg/dl<br>mmol/l	0.5 (-2.5, 3.6)<br>0.01 (-0.06, 0.09)	0.725	6.8 (2.9, 10.6)<br>0.17 (0.07, 0.27)	0.001	1.5 (-1.2, 4.1)<br>0.04 (-0.01, 0.11)	0.273
eGFR, ml/min/1.73 m^2	1.5 (-0.02, 3.0)	0.052	2.4 (0.2, 4.7)	0.035	-0.6 (-2.6, 1.4)	0.549
UACR, mg/g<br>mg/mmol	11.0 (-3.9, 25.9)<br>1.2 (-0.4, 2.9)	0.146	0.1 (-49.3, 49.6)<br>0.0 (-5.6, 5-6)	0.996	-11.8 (-41.2, 17.6)<br>-1.3 (4.7, 2.0)	0.433

ESM Tab. 4. Characteristics of participants by diabetes remission status.

	Cohort 1				Cohort 2			
	**All**	**Remission**	**No remission**	**p-value**	**All**	**Remission**	**No remission**	**p-value**
**Number**	1934	145	1706		13277	649	10557	
**Age, years**	62.7 (11.7)	61.6 (11.3)	62.8 (11.7)	0.266	61.6 (12.7)	60.8 (12.0)	61.7 (12.6)	0.076
**Male, %**	57.3	60.0	56.7	0.447	58.9	60.6	58.4	0.283
**Diabetes duration, years**	0.8 (1.8)	0.4 (0.6)	0.8 (1.8)	0.008	1.0 (1.3)	0.5 (1.1)	1.0 (1.4)	<0.001
**Body mass index, kg/m^2**	29.5 (5.2)	29.5 (4.7)	29.6 (5.2)	0.894	30.1 (5.7)	30.8 (6.1)	30.2 (5.7)	0.009
**Lab & risk factors**								
**HbA_{1c}, mmol/mol**<br>**%**	64 (17)<br>8.0 (2.1)	64 (16)<br>8.0 (2.0)	65 /17)<br>8.1 (2.1)	0.577	57 (13)<br>7.4 (1.7)	62 (14)<br>7.8 (1.7)	59 (13)<br>7.6 (1.7)	0.001
**Systolic blood pressure, mm Hg**	140.5 (20.3)	139.0 (22.5)	141.0 (20.2)	0.270	137.8 (19.4)	139.5 (19.3)	137.9 (19.5)	0.041
**Diastolic blood pressure, mm Hg**	83.1 (10.7)	83.4 (11.7)	83.2 (10.7)	0.875	80.7 (10.6)	82.0 (10.6)	80.8 (10.6)	0.008
**Total cholesterol, mg/dl**<br>**mmol/l**	206.8 (48.3)<br>5.3 (1.7)	213.7 (48.5)<br>5.5 (1.7)	206.9 (48.7)<br>5.3 (1.7)	0.172	189.1 (43.3)<br>4.8 (1.5)	193.2 (43.1)<br>5.0 (1.5)	190.0 (43.4)<br>4.9 (1.5)	0.087
**HDL cholesterol, mg/dl**<br>**mmol/l**	49.8 (14.9)<br>1.3 (0.5)	48.5 (15.5)<br>1.2 (0.5)	49.9 (14.9)<br>1.3 (0.5)	0.376	49.0 (13.7)<br>1.3 (0.5)	48.1 (13.2)<br>1.2 (0.5)	48.8 (13.7)<br>1.3 (0.5)	0.239
**LDL cholesterol, mg/dl**<br>**mmol/l**	124.5 (39.6)<br>3.2 (1.4)	129.0 (40.0)<br>3.3 (1.4)	124.5 (39.9)<br>3.2 (1.4)	0.314	110.7 (36.9)<br>2.8 (1.3)	113.5 (36.2)<br>2.9 (1.2)	111.0 (37.0)<br>2.8 (1.3)	0.138
**Triglycerides, mg/dl**<br>**mmol/l**	150.0 (77.0)<br>1.7 (0.9)	163.7 (82.4)<br>1.8 (0.9)	150.4 (76.9)<br>1.7 (0.9)	0.119	148.0 (112.2)<br>1.7 (1.3)	152.9 (100.7)<br>1.7 (1.1)	152.2 (118.6)<br>1.7 (1.3)	0.884
**eGFR, ml/min/1.73 m^2**	81.0 (19.1)	81.3 (20.9)	81.2 (18.8)	0.937	83.7 (22.0)	83.1 (20.5)	84.1 (22.1)	0.307
**UACR, mg/g**	43.0 (137.2)	41.6 (90.5)	44.3 (144.4)	0.912	51.6 (223.9)	41.7 (107.6)	53.5 (229.9)	0.630
**Complications**								
**eGFR<60 ml/min/1.73 m^2**	14.1	13.8	13.8	0.995	10.3	10.9	9.9	0.404
**UACR >30 mg/g**	20.2	20.0	25.7	0.422	5.3	2.8	5.9	<0.001
**Retinopathy, %**	18.7	0.0	19.5	0.122	1.3	0.8	1.4	0.184
**Cardiovascular disease, %**	2.4	0.0	2.6	0.050	2.6	1.8	2.7	0.170
**Macrovascular disease, %**	4.2	0.0	4.5	0.009	16.4	14.0	16.6	0.081
**Microvascular disease, %**	37.9	37.5	38.3	0.911	7.4	3.9	7.8	<0.001
**Comorbidities**								
**History of cancer, %**	5.7	6.9	5.5	0.466	N/A	N/A	N/A	N/A
**Inflammatory diseases, %**	12.2	13.8	12.0	0.516	N/A	N/A	N/A	N/A
**RxRisk comorbidity index**	4.7 (5.1)	4.3 (5.2)	4.7 (5.1)	0.433	4.0 (5.4)	4.2 (5.9)	3.9 (5.4)	0.217
**Diabetes therapy**								
**Metformin, %**	43.6	23.4	46.9	<0.001	50.5	35.4	56.5	<0.001
**Sulphonylurea, %**	24.3	9.0	26.4	<0.001	6.7	2.2	7.9	<0.001
**DPP-4 inhibitors, %**	1.8	0.7	1.9	0.284	3.5	0.3	4.3	<0.001

**GLP-1 receptor agonists, %**	0.7	0.0	0.8	0.274	0.7	0.5	0.9	0.270
**SGLT2 inhibitors, %**	0.3	0.0	0.2	0.560	0.6	0.0	0.7	0.028
**Thiazolidinediones, %**	0.3	0.0	0.4	0.475	0.8	0.5	0.9	0.279
**Bolus insulin, %**	11.1	8.3	11.8	0.204	4.6	2.0	5.4	<0.001
**Basal insulin, %**	6.7	5.5	7.0	0.490	4.9	1.1	6.0	<0.001
**Other therapies**								
**Statins, %**	25.6	20.7	26.1	0.153	37.8	33.3	38.7	0.006
**Anti-platelet agents, %**	51.8	49.0	51.8	0.510	25.8	23.7	25.8	0.245
**RAS blockers, %**	41.0	43.4	40.9	0.552	45.0	46.8	45.2	0.405
**Beta blockers, %**	16.6	15.9	16.8	0.766	20.3	21.9	20.0	0.253
**Calcium channel blockers, %**	16.6	16.6	16.5	0.995	15.0	14.9	15.0	0.947
**Diuretics, %**	28.1	33.1	27.4	0.144	29.2	32.5	29.0	0.054

ESM Tab. 5. Characteristics of Cohort 1 divided in three groups based on weight loss. Group 0: <5% weight loss. Group 1: 5% to <10% weight loss, group 2: 10% or more weight loss. p1 indicates the p-values for the comparison of group 1 vs group 0, p2 indicates the p-values for the comparison between group 2 vs group 0, p3 indicates the p-values for the comparison of group 1 vs group 2.

	Group 2	Group 1	Group 0	p1	p2	p3
**Number**	308	545	1081			
**Demographics**						
**Female, %**	50.3	46.4	38.7	0.003	<0.001	0.274
**Age, years**	62.0 (12.5)	63.7 (12.0)	62.4 (11.2)	0.044	0.513	0.050
**Diabetes duration, years**	0.8 (1.8)	0.8 (1.6)	0.8 (1.8)	0.726	0.743	0.962
**Risk factors and lab**						
**Body mass index, kg/m^2**	31.1 (5.8)	30.0 (5.4)	28.7 (4.7)	<0.001	<0.001	0.007
**Lab & risk factors**						
**HbA_{1c}, mmol/mol**<br>**%**	66 (17)<br>8.2 (2.1)	64 /16)<br>8.0 (2.0)	63 (18)<br>7.9 (2.2)	0.326	0.035	0.201
**Systolic blood pressure, mm Hg**	141.2 (21.7)	141.2 (19.6)	140.0 (20.2)	0.238	0.371	0.967
**Diastolic blood pressure, mm Hg**	83.9 (11.8)	83.0 (10.2)	82.9 (10.7)	0.854	0.183	0.276
**Total cholesterol, mg/dl**<br>**mmol/l**	208.8 (51.5)<br>5.4 (1.8)	207.6 (45.5)<br>5.3 (1.6)	205.8 (48.9)<br>5.3 (1.7)	0.548	0.434	0.766
**HDL cholesterol, mg/dl**<br>**mmol/l**	49.3 (14.2)<br>1.3 (0.5)	48.7 (14.6)<br>1.2 (0.5)	50.5 (15.2)<br>1.3 (0.5)	0.063	0.303	0.655
**LDL cholesterol, mg/dl**<br>**mmol/l**	123.6 (41.3)<br>3.2 (1.4)	126.4 (39.1)<br>3.2 (1.3)	123.9 (39.3)<br>3.2 (1.4)	0.349	0.942	0.459
**Triglycerides, mg/dl**<br>**mmol/l**	158.6 (91.1)<br>1.8 (1.0)	158.2 (79.2)<br>1.8 (0.9)	143.4 (70.7)<br>1.6 (0.8)	0.003	0.018	0.962
**eGFR, ml/min/1.73 m^2**	82.3 (21.3)	79.2 (18.8)	81.6 (18.5)	0.061	0.651	0.083
**UACR, mg/g**<br>**mg/mmol**	25.6 (56.1)<br>2.9 (6.3)	48.1 (157.0)<br>5.4 (17.8)	45.7 (142.9)<br>5.2 (16.2)	0.877	0.228	0.233
**Complications**						
**Cardiovascular disease, %**	2.9	2.6	2.1	0.574	0.413	0.760
**Coronary artery disease, %**	2.6	2.2	2.0	0.825	0.550	0.714
**Heart failure, %**	0.0	8.8	2.5	0.040	0.335	0.065
**Atrial fibrillation, %**	0.6	0.4	1.3	0.074	0.349	0.563
**Macrovascular disease, %**	4.2	4.0	4.3	0.835	0.979	0.897
**CKD, %**	19.6	23.7	19.9	0.144	0.897	0.237
**eGFR<60 ml/min/1.73 m^2**	14.4	16.8	12.5	0.061	0.472	0.458
**Microvascular disease, %**	35.9	38.2	38.3	0.978	0.631	0.670
**Retinopathy, %**	13.8	15.0	21.3	0.304	0.360	0.882
**Cerebrovascular disease, %**	0.3	0.4	0.1	0.224	0.344	0.920
**Carotid atherosclerosis, %**	1.0	2.2	2.3	0.888	0.141	0.190
**Peripheral arterial disease, %**	50.0	22.2	66.7	0.046	0.679	0.476
**Comorbidities**						
**History of cancer, %**	8.1	5.3	5.2	0.904	0.052	0.108

**Inflammatory diseases, %**	17.2	12.1	10.7	0.405	0.002	0.039
**RxRisk comorbidity index**	5.8 (6.5)	4.4 (4.9)	4.5 (4.7)	0.904	<0.001	<0.001
**Diabetes therapy**						
**Metformin, %**	50.3	42.4	42.4	0.995	0.013	0.025
**Sulphonylurea, %**	16.2	23.3	27.1	0.098	<0.001	0.014
**Glitazones, %**	0.0	0.2	0.5	0.381	0.232	0.453
**SGLT2 inhibitors, %**	0.6	0.0	0.3	0.219	0.337	0.060
**GLP-1RA, %**	0.6	1.1	0.6	0.225	0.847	0.512
**DPP-4 inhibitors, %**	2.9	2.0	1.3	0.263	0.048	0.403
**Bolus insulin, %**	7.1	9.9	12.9	0.083	0.006	0.174
**Basal insulin, %**	6.8	6.2	6.9	0.595	0.942	0.741
**Any insulin, %**	10.7	11.6	15.4	0.038	0.040	0.708
**Other therapies**						
**RAS blockers, %**	43.2	41.8	40.0	0.468	0.311	0.703
**Calcium channel blockers, %**	16.9	17.2	16.3	0.621	0.802	0.892
**Alpha blockers, %**	4.2	4.6	4.1	0.626	0.907	0.804
**Beta blockers, %**	17.9	16.9	16.1	0.687	0.463	0.717
**Anti-platelet, %**	54.2	53.4	50.2	0.229	0.217	0.816
**Diuretics, %**	32.1	28.6	26.6	0.397	0.058	0.281
**Statins, %**	26.9	23.1	26.5	0.144	0.863	0.212

eGFR, estimated glomerular filtration rate. UACR, urinary albumin creatinine ratio. CKD, chronic kidney disease. RAS, renin angiotensin system.

ESM Tab. 6. Characteristics of cohort 2 divided in three groups based on weight loss. Group 0: <5% weight loss. Group 1: 5% to <10% weight loss, group 2: 10% or more weight loss. p1 indicates the p-values for the comparison of group 1 vs group 0, p2 indicates the p-values for the comparison between group 2 vs group 0, p3 indicates the p-values for the comparison of group 1 vs group 2.

	Group 2	Group 1	Group 0	p1	p2	p3
**Number**	1167	2304	9806			
**Demographics**						
**Age, years**	61.4 (13.2)	62.1 (12.8)	61.5 (12.6)	0.046	0.758	0.129
**Sex male %**	49.3	55.3	60.9	<0.001	<0.001	<0.001
**Diabetes duration, years**	0.5 (1.0)	0.7 (1.2)	1.1 (1.4)	<0.001	<0.001	<0.001
**Risk factors and lab**						
**Weight, kg**	88.4 (20.8)	85.7 (18.5)	82.8 (17.2)	<0.001	<0.001	<0.001
**Body mas index, kg/m^2**	32.0 (6.4)	30.8 (5.8)	29.7 (5.5)	<0.001	<0.001	<0.001
**Waist cm**	107.6 (13.7)	105.8 (13.2)	103.1 (12.7)	<0.001	<0.001	0.002
**Systolic blood pressure, mm Hg**	139.2 (20.5)	139.6 (19.2)	137.3 (19.4)	<0.001	0.002	0.566
**Diastolic blood pressure, mm Hg**	81.3 (11.0)	81.5 (10.5)	80.5 (10.5)	<0.001	0.026	0.489
**Fasting glucose, mg/dl**	150.0 (56.3)	150.8 (53.3)	145.7 (54.7)	<0.001	0.012	0.687
**HbA$_{1c}$, mmol/mol**<br>**%**	57 (14)<br>7.4 (1.8)	58 (12)<br>7.5 (1.6)	56 (13)<br>7.3 (1.7)	<0.001	<0.001	0.069
**Total cholesterol, mg/dl**<br>**mmol/l**	189.1 (44.0)<br>4.8 (1.5)	192.3 (43.6)<br>4.9 (1.5)	187.8 (43.1)<br>4.8 (1.5)	<0.001	<0.001	0.417
**HDL cholesterol, mg/dl**<br>**mmol/l**	49.0 (14.2)<br>1.3 (0.5)	48.4 (13.4)<br>1.2 (0.5)	49.2 (13.7)<br>1.3 (0.5)	0.018	0.059	0.884
**Triglycerides, mg/dl**<br>**mmol/l**	148.1 (120.7)<br>1.7 (1.3)	156.9 (120.8)<br>1.8 (1.3)	144.7 (108.8)<br>1.6 (1.2)	<0.001	<0.001	0.695
**LDL cholesterol, mg/dl**<br>**mmol/l**	110.7 (37.0)<br>2.8 (1.3)	112.9 (37.7)<br>2.9 (1.3)	109.8 (36.6)<br>2.8 (1.3)	0.001	0.002	0.518
**eGFR, ml/min/1.73 m^2**	83.7 (21.6)	82.6 (23.8)	84.0 (21.5)	0.011	0.246	0.532
**Albuminuria, mg/g**<br>**mg/mmol**	51.7 (263.0)<br>5.8 (29.7(	71.8 (289.0)<br>8.1 (32.7)	45.4 (200.3)<br>5.1 (22.6)	0.008	0.114	0.740
**Complications**						
**CKD stage III+, %**	11.0	11.3	9.9	0.052	0.261	0.780
**Pathologic albuminuria, %**	6.2	6.1	5.1	0.040	0.081	0.875
**Retinopathy, %**	0.8	1.2	1.4	0.498	0.129	0.338
**Peripheral arterial disease, %**	0.8	0.9	0.9	0.718	0.551	0.767
**Stroke / TIA, %**	0.0	0.0	0.2	0.060	0.181	N/D
**Ischemic heart disease, %**	2.5	1.8	2.1	0.365	0.419	0.193
**Heart failure, %**	0.2	0.1	0.2	0.392	0.948	0.488
**Any site revascularization, %**	1.4	0.9	1.4	0.075	0.987	0.213
**Microangiopathy, %**	17.5	17.7	16.0	0.045	0.145	0.967
**Macroangiopathy, %**	5.0	6.2	8.0	0.004	<0.001	0.171
**Established cardiovascular disease, %**	2.7	2.3	2.7	0.266	0.953	0.427
**RxRisk**	4.5 (6.0)	4.1 (5.3)	3.9 (5.3)	0.087	<0.001	0.040

**Diabete therapy**						
**Metformin, %**	50.3	51.3	50.3	0.404	0.975	0.610
**Sulphonylurea, %**	5.7	5.6	7.0	0.016	0.076	0.987
**DPP-4 inhibitors, %**	2.2	2.3	3.9	<0.001	0.004	0.893
**GLP-1RA, %**	0.9	0.9	0.7	0.181	0.256	0.928
**SGLT2 inhibitors, %**	0.5	0.6	0.6	0.973	0.713	0.731
**Pioglitazone, %**	0.6	0.4	0.8	0.041	0.567	0.330
**Acarbose, %**	0.2	0.1	0.1	0.923	0.658	0.763
**Bolus insulin, %**	4.9	4.0	4.7	0.196	0.636	0.203
**Basal insulin, %**	3.8	4.3	5.2	0.054	0.032	0.497
**Other therapies**						
**Statin, %**	33.8	35.9	38.7	0.011	0.001	0.253
**Anti-platelet agents, %**	22.1	25.9	26.2	0.793	0.004	0.019
**RAS blockers, %**	43.7	46.9	44.8	0.069	0.484	0.076
**Beta blockers, %**	21.2	21.0	20.1	0.353	0.392	0.890
**Calcium channel blockers, %**	14.0	15.6	14.9	0.431	0.426	0.234
**Diuretics, %**	29.1	31.8	28.6	0.002	0.700	0.107

eGFR, estimated glomerular filtration rate. UACR, urinary albumin creatinine ratio. CKD, chronic kidney disease. RAS, renin angiotensin system.

ESM Tab. 7. Three-group analysis of the change over time of risk factors in over the observation period.

A) Cohort 1

Variable	Group 1 vs 0	Group 2 vs 0	Group 2 vs 1
Primary endpoint			
HbA_{1c}, mmol/mol<br>%	-1.3 (0.3)*<br>-0.12 (0.03)*	-2.4 (0.5)*<br>-0.22 (0.05)*	-1.2 (0.5)*<br>-0.11 (0.05)*
Secondary endpoints			
Body weight, kg	-3.4 (0.3)*	-7.0 (0.4)*	-3.6 (0.4)*
Systolic blood pressure, mm Hg	-1.4 (1.0)	-1.6 (1.1)	-3.0 (1.2)*
HDL cholesterol, mg/dl<br>mmol/l	-1.3 (0.8)<br>-0.03 (0.03)	1.7 (1.0)<br>0.04 (0.03)	3.0 (1.0)*<br>0.08 (0.03)
Triglycerides, mg/dl<br>mmol/l	-5.1 (4.4)<br>0.1 (0.0)	-3.3 (4.9)<br>0.0 (0.1)	1.8 (5.1)<br>0.0 (0.1)
LDL cholesterol, mg/dl<br>mmol/l	0.1 (1.9)<br>0.00 (0.07)	5.4 (2.2)*<br>0.14 (0.06)*	5.3 (2.3)*<br>0.14 (0.06)*
eGFR, ml/min/1.73 m^2	-0.9 (1.2)	1.8 (1.4)	2.7 (1.5)
UACR, mg/g<br>mg/mmol	6.3 (23.4)<br>0.7 (2.6)	-3.1 (26.0)<br>-0.4 (2.9)	-9.4 (28.6)<br>-1.1 (3.2)

B) Cohort 2

Variable	Group 1 vs 0	Group 2 vs 0	Group 2 vs 1
Primary endpoint			
HbA_{1c}, mmol/mol<br>%	-1.4 (0.4)*<br>-0.13 (0.04)*	-3.1 (0.5)*<br>-0.28 (0.05)*	-1.6 (0.5)*<br>-0.15 (0.05)*
Secondary endpoints			
Body weight, kg	-4.4 (0.4)*	-8.6 (0.5)*	-4.2 (0.5)*
Systolic blood pressure, mm Hg	-1.6 (0.8)*	-4.8 (1.0)*	-3.1 (1.0)*
HDL cholesterol, mg/dl<br>mmol/l	0.8 (0.3)*<br>0.02 (0.01)*	2.2 (0.4)*<br>0.06 (0.01)*	1.3 (0.4)*<br>0.03 (0.01)*
Triglycerides, mg/dl<br>mmol/l	-7.5 (2.7)*<br>-0.1 (0.0)*	-17.8 (3.7)*<br>-0.2 (0.0)*	-10.4 (3.6)*<br>-0.1 (0.0)*
LDL cholesterol, mg/dl<br>mmol/l	-2.6 (1.1)*<br>-0.07 (0.03)*	-2.5 (1.4)<br>-0.06 (0.04)*	0.1 (1.4)<br>0.00 (0.01)
eGFR, ml/min/1.73 m^2	0.1 (0.5)	-0.1 (0.7)	-0.2 (0.6)
UACR, mg/g<br>mg/mmol	32.4 (20.7)<br>3.7 (2.3)	1.9 (18.2)<br>0.2 (2.1)	-30.5 (20.7)<br>-3.4 (2.3)

Group 0: weight loss less than 5%, Group 1: weight loss 5% to <10%, Group 2: weight loss 10% or more.
**p<0.05 after correction for multiple comparisons.*

ESM Fig. 1. Study flowchart. a) Timeline of the two cohorts and windows of exposure (EHR, electronic health record). b) Number of patients and duration of observation in the two cohorts.

a

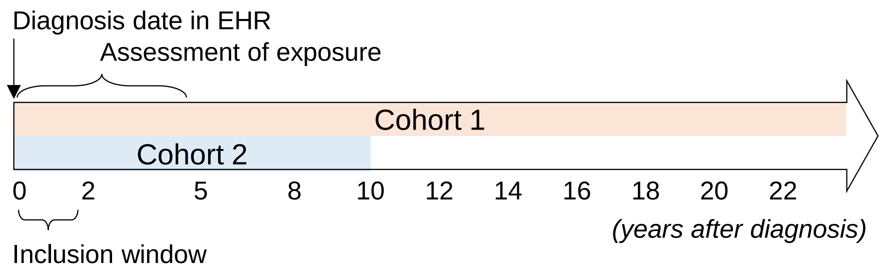

b

Step	Cohort 1	Cohort 2
Total, n	11,254	145,198
Included within 2 years after diagnosis, n	3970	50,080
Available data for assessment of exposure, n	3902	29,536
Available data for primary outcome, n	1934	13,277
Median observation, years	10.8	4.0
Max follow-up, years	24.0	8.5

ESM Fig. 2. Weight loss and burden of therapy. a, b) The burden of therapy, defined as the number of glucose lowering medication (GLM) classes over time, is reported for the two groups of cohort 1 (a) and cohort 2 (b). The rates of insulin initiation analysed using the Cox regression model in cohort 1 (c) and cohort 2 (d). There were no missing data for these outcomes.

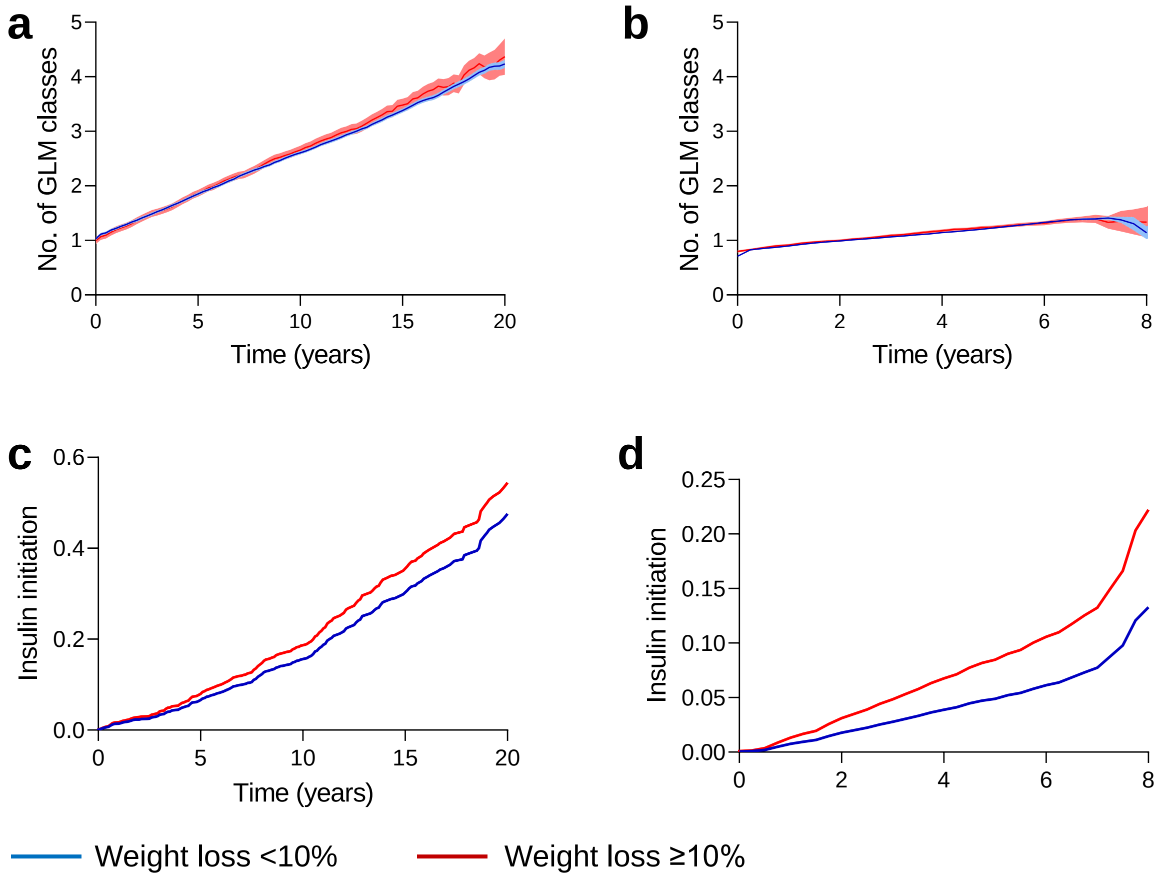

ESM Fig. 3. Weight loss and cardio-metabolic parameters. For cohort 1 (a, c, e) and cohort 2 (b, d, f), the change over time in systolic blood pressure (SBP), triglycerides, and HDL cholesterol are shown for the to groups. The number of patients with available data for each outcome is reported.

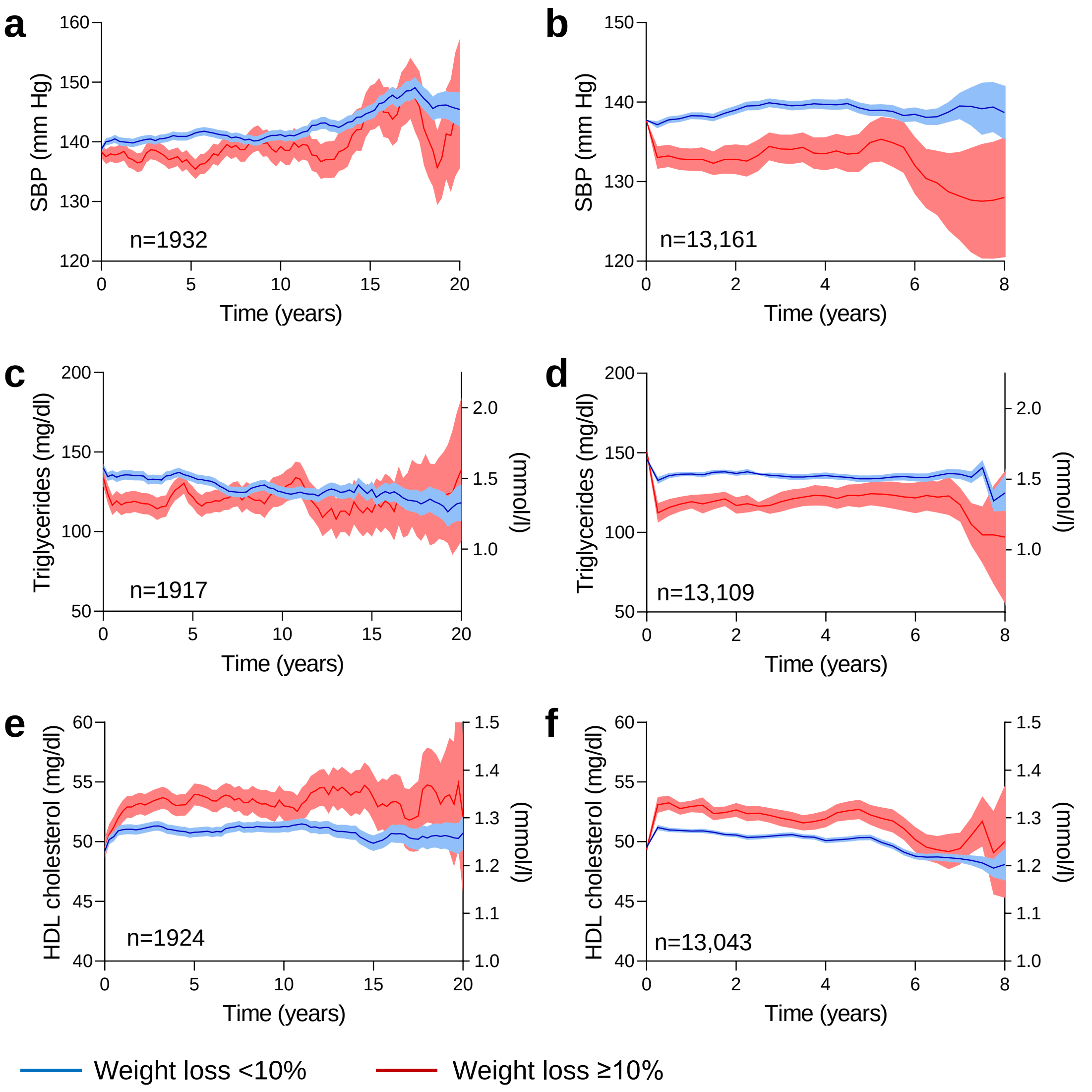

ESM Fig. 4. Weight loss and markers of kidney function and damage. For cohort 1 (a, c) and cohort 2 (b, d), the estimated glomerular filtration rate (eGFR) and the urinary albumin-to-creatinine ratio (UACR) are shown for both groups. The number of patients with available data for each outcome is reported.

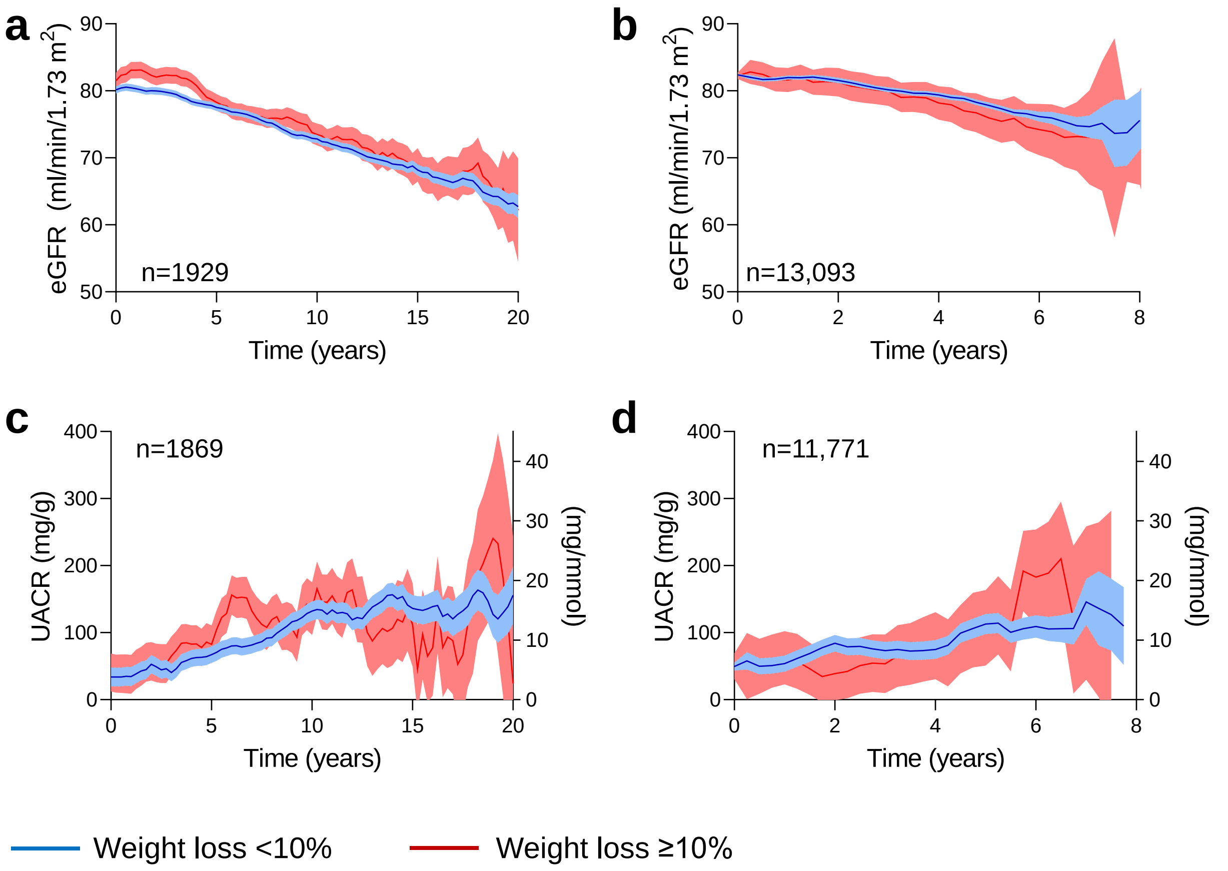

ESM Fig. 5: Weight over time by weight loss group and remission status. Participants in Cohort 1 (a,b) and Cohort 2 (c,d) were divided into 4 groups based on whether or not they lost ≥10% body weight and on whether or not they experienced remission within the first 5 years after diagnosis. For both cohorts, weight curves (a,c) are derived from an adjusted MMRM, while histograms (b,d) display the mean estimated difference in the entire period of observation (bars indicate standard error of the mean).

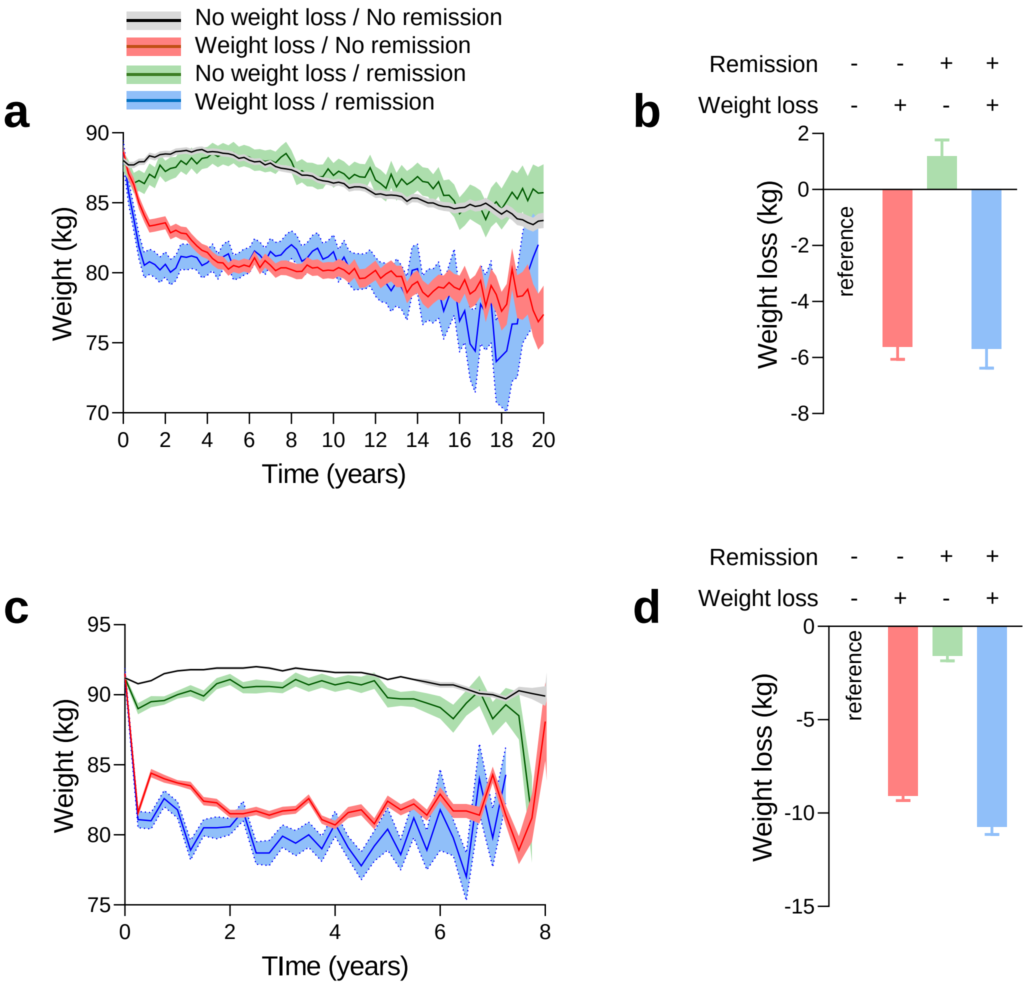

ESM Fig. 6. Main outcomes of participants in the two cohorts divided in 3 groups based on percentage of body weight. Data are presented separately for Cohort 1 (a-c) and Cohort 2 (d-f). a,d) Changes over time in body weight. b,e) Changes in HbA1c, c,f) incidence of remission: adjusted hazard ratios (HR) are reported versus group 0 (reference) with their 95% confidence intervals.\

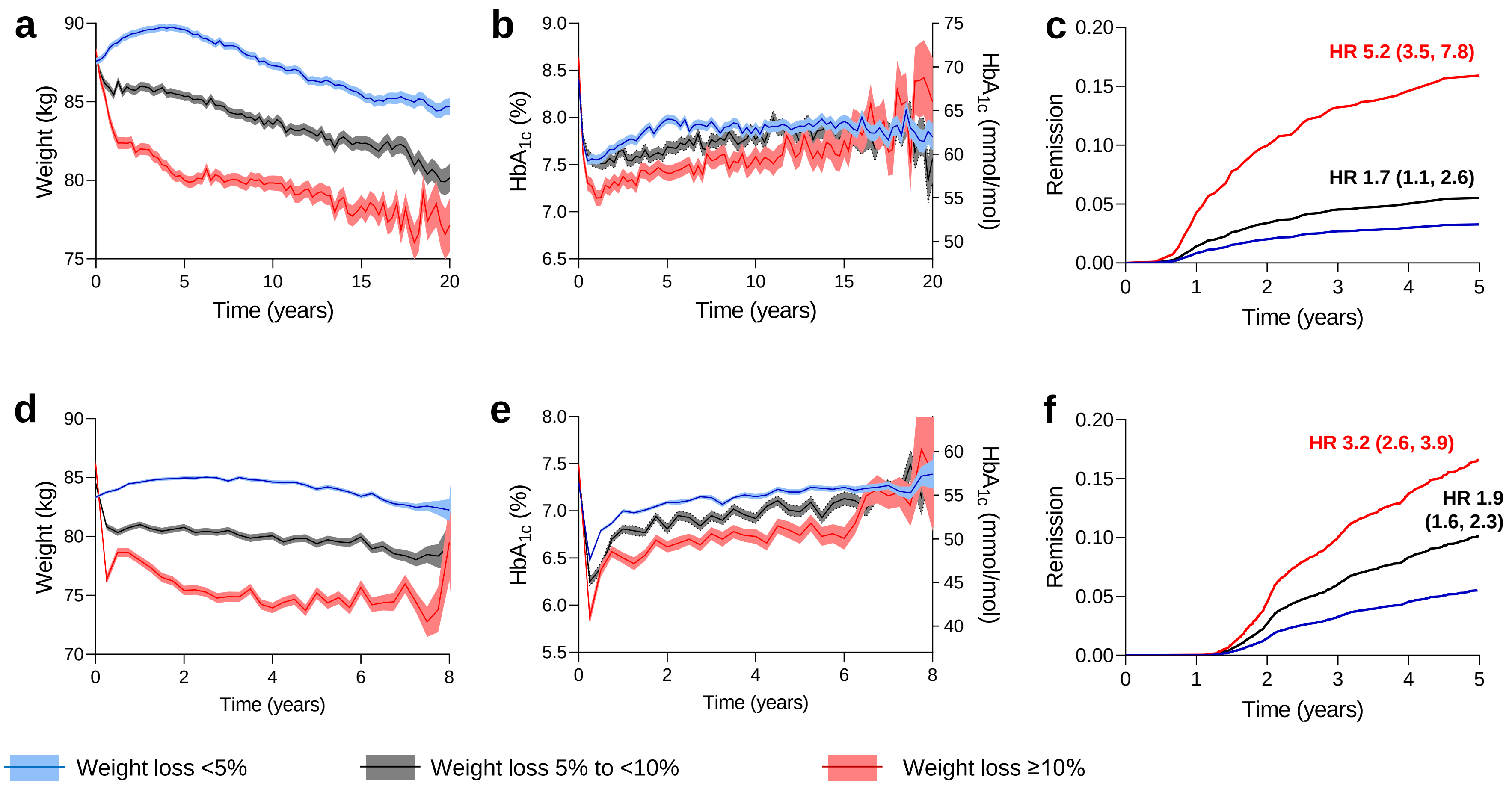

Supplement: Supplementary file 1 — ESM (PDF 1034 KB) [file 125_2025_6402_MOESM1_ESM.pdf]
